# Effective Dose Range of Intrathecal Isobaric Bupivacaine to Achieve T5–T10 Sensory Block Heights for Elderly and Overweight Patients: An Observational Study

**DOI:** 10.3390/medicina59030484

**Published:** 2023-03-01

**Authors:** Ornwara Visavakul, Prangmalee Leurcharusmee, Tanyong Pipanmekaporn, Jiraporn Khorana, Jayanton Patumanond, Phichayut Phinyo

**Affiliations:** 1 Faculty of Medicine, Chiang Mai University, Chiang Mai 50200, Thailand; 2 Department of Anesthesiology, Faculty of Medicine, Chiang Mai University, Chiang Mai 50200, Thailand; 3 Center for Clinical Epidemiology and Clinical Statistics, Faculty of Medicine, Chiang Mai University, Chiang Mai 50200, Thailand; 4 Department of Surgery, Faculty of Medicine, Chiang Mai University, Chiang Mai 50200, Thailand; 5 Department of Family Medicine, Faculty of Medicine, Chiang Mai University, Chiang Mai 50200, Thailand; 6 Musculoskeletal Science and Translational Research (MSTR), Chiang Mai University, Chiang Mai 50200, Thailand

**Keywords:** aged, bupivacaine, drug dose–response relationship, obesity, overweight, spinal anesthesia

## Abstract

*Background and Objectives:* The dose selection for isobaric bupivacaine determines the success of spinal anesthesia (SA). A dose higher than the optimal dose causes high SA, whereas an underdose leads to inadequate spread of cephalad. As it involves anatomical and physiological alterations, the dosing should be reduced with advancing age and body mass index values. Therefore, this study aimed to demonstrate the association between the isobaric bupivacaine dose and block height, and to determine the dose intervals of bupivacaine to achieve the T5–T10 sensory block with a low probability of high SA in elderly and overweight patients. *Material and Methods:* This retrospective observational study recruited 1079 adult patients who underwent SA with 0.5% isobaric bupivacaine from 2018 to 2021. The patients were divided into four categories: category 1 (age < 60, BMI < 25), category 2 (age < 60, BMI ≥ 25), category 3 (age ≥ 60, BMI < 25), and category 4 (age ≥ 60, BMI ≥ 25). The bupivacaine dose and sensory block height (classified into three levels: high (T1–T4), favorable (T5–T10), and low (T11–L2)) were recorded. *Results:* The sensory block level increased significantly with increasing doses of bupivacaine for patients in categories 1 and 2. The suggested dose ranges for the favorable block heights were 15–17 and 10.5–16 mg in patient categories 1–2 and 3–4, respectively. In these dose ranges, the probability range of high SA was 10–15%. *Conclusions:* The sensory block height following SA was associated with the bupivacaine dose in patients aged <60 years. Regardless of the BMI, the suggested dose ranges of 0.5% isobaric bupivacaine are 15–17 mg (3.0–3.4 mL) and 10.5–16 mg (2.1–3.2 mL) for patients aged <60 and ≥60 years, respectively.

## 1. Introduction

Spinal anesthesia (SA) is routinely performed in surgical procedures that require anesthetic coverage below the T4 spinal level [1,2]. One of the commonly used local anesthetics for SA is bupivacaine hydrochloride. ‘Hyperbaric’ and ‘isobaric’ solutions are two forms of commercial bupivacaine. In contrast to its hyperbaric counterpart, isobaric bupivacaine is not influenced by gravitational forces, meaning its intrathecal spread or block height after a single-shot injection depends substantially on the dose of the administered drug [3,4].

The dose selection for isobaric bupivacaine determines the success of the SA. The recommended doses to reach the spinal block height at T4 and T10 in adults are 12–20 and 10–15 mg, respectively [1]. As it involves anatomical and physiological alterations, the dosing should be reduced with advancing age and body mass index (BMI) values [5,6]. With the aging process, there are configurational changes in the spinal column and a decreased total cerebrospinal fluid (CSF) volume [7,8]. With obesity, increases in abdominal and epidural fat reduce the lumbosacral CSF volume [8,9,10,11,12]. These variations are attributed to a greater degree of cephalad spread of SA. An intrathecal isobaric bupivacaine dose higher than the optimal dose causes a high spinal block, whereas an underdose leads to inadequate cephalad spread (i.e., failed spinal block) [13,14]. High and failed blocks are problematic, especially in elderly and overweight patients. A sensory block height higher than T6 is an independent risk factor for hypotension, bradycardia, and reduced stroke volume after SA [15,16,17]. Accordingly, a dosage guide of isobaric bupivacaine for SA allows the potential prevention of these undesired events.

This retrospective study aimed to demonstrate the association between the dose of intrathecal isobaric bupivacaine and the sensory block height and to determine the dose ranges of isobaric bupivacaine for a single-shot intrathecal injection in general adult, overweight, elderly, and elderly overweight patients. We hypothesized that the doses of isobaric bupivacaine to achieve the T5–T10 sensory block, which is a ‘favorable’ block height for frequently performed operations, in patients aged ≥60 years or BMI ≥ 25 kg/m^2^ are lower than that of normal adult patients.

## 2. Materials and Methods

### 2.1. Study Population

After ethical approval by the Research Ethics Committee of the Faculty of Medicine, Chiang Mai University on 15 December 2021 (study code: ANE-2564-09649) and clinical trial registration (thaiclinicaltrials.org: TCTR20220508002) on 8 May 2022, the data for adult patients undergoing SA with 0.5% isobaric bupivacaine for any surgical procedure from January 2018 to December 2021 were collected retrospectively. Informed consent was waived by the Research Ethics Committee owing to the retrospective nature of the data collection. All collected data were kept confidential and accessible only to investigators. The exclusion criteria for data collection included parturient, failed, or repeated SA; the co-administration of intrathecal adjuvants; and incomplete records of the bupivacaine dose, sensory block height, operative site, and lumbar puncture level.

Patients were divided into four categories according to age and BMI. These two parameters are both independent predictors of an exaggerated block height after SA because the lumbosacral CSF volume is smaller in those who have increased BMI or spinal stenosis [8]. In overweight and obese patients (BMI ≥ 25 kg/m^2^), the peak sensory block level following SA was higher than in normal adults (BMI 18.5–24.9 kg/m^2^) [18]. Moreover, the prevalence of acquired lumbar spinal stenosis is higher at ages ≥ 60 years [19]. Therefore, in our study, the pre-selected cut-off point for age was 60 years, and that for BMI was 25 kg/m^2^.

### 2.2. Spinal Block Technique

As per the traditional technique used in our institute, spinal blocks were performed quite uniformly. Standard anesthetic monitoring was applied, and intravenous fluid pre-loading or co-loading was administered. A patient was placed in the lateral decubitus position, while an anesthesia resident performed the procedure under the supervision of an attending anesthesiologist. The size of the Quincke spinal needle (25G or 27G), injection level estimated from the Tuffier’s line (at the L2–S1 levels), needle approach (midline or paramedian), and dose (or volume) of 0.5% isobaric bupivacaine were chosen at the discretion of the attending anesthesiologists. To ensure a full dose for the intrathecal drug administration, the bupivacaine was incrementally injected, and the free aspirated CSF flow was confirmed every 1 mL of injected bupivacaine. If the CSF did not flow freely, the spinal needle was rotated or repositioned. Generally, the rate of injection was approximately 0.1–0.2 mL/s. No intentional aspiration or reinjection of CSF (i.e., barbotage technique) was performed. At the end of the intrathecal injection, the patient lay supine, and the level of the sensory block on the midclavicular line was assessed using a cold pack or pinprick within 30 min. The highest dermatome at which there was a decreased sensation of cold or pain was recorded as the block height.

### 2.3. Outcome Measurements

The characteristic data included the sex, age, weight, height, BMI, and type of operation. The procedural data included the dose of isobaric bupivacaine, level of lumbar puncture, and peak sensory block level.

The sensory block levels were classified into three anesthetic outcomes: ‘high’, ‘favorable’, and ‘low’ dermatomal levels of sensory blocks. A high block level represented sensory block heights as high as T1–T4, which are typically related to hemodynamic instability [15,17]. The favorable sensory block level represented an anesthetic coverage up to the T5–T10 dermatomal levels, which are adequate anesthetic levels for abdominal (e.g., intestinal, urologic, gynecologic surgery), inguinal hernia, pelvic, and hip surgeries [1,2]. The low block height represented peak sensory block levels at T11–L2, which are sufficient for ankle, foot, perineal, and perianal surgeries [1,2]. This study focused on the association between the dose intervals of isobaric bupivacaine given to achieve the ‘favorable’ block height to avoid high SA or an inadequate spinal block for frequently performed operations. The suggested dose interval of isobaric bupivacaine to increase the probability of achieving a favorable block height while reducing the probability of a high sensory block was determined based on the average and half of the average incidence rate of high SA.

### 2.4. Statistical Analysis

All statistical analyses were conducted using Stata 17 software (StataCorp, College Station, TX, USA). Continuous variables were described using mean and standard deviation (SD) values and compared using a one-way analysis of variance (ANOVA). Categorical variables were described using the frequency and percentage and compared using Fisher’s exact probability test or a Chi-square test, as appropriate.

To examine the association between the isobaric bupivacaine dose and sensory block levels, a multivariable polytomous logistic regression was performed separately for each of the four subcategories of patients. The potential determinants incorporated within the model were the dose of isobaric bupivacaine, sex, age, height, weight, and level of lumbar puncture. Instead of incorporating the BMI as a single anthropometric measurement within our model, we separately modeled both the height and weight as predictors of block levels to preserve detailed information. A favorable sensory block level was defined as the base outcome in the polytomous logistic model. The linearity assumption of the association between the continuous determinants and outcome was inspected using a multivariable fractional polynomial (MFP) algorithm. In our analysis, the MFP algorithm revealed that all continuous determinants could be adequately fitted in their linear forms.

We predicted the probabilities of achieving low, favorable, or high sensory block levels for each 0.5-mg increment in intrathecal isobaric bupivacaine from 5 to 20 mg, and illustrated these probabilities using marginal prediction curves. As the primary goal was to reduce the likelihood of achieving a high sensory block level, we aimed to determine the recommended dose interval of isobaric bupivacaine based on the following pre-specified criteria. First, the lower boundary of the suggested dose was located at the dose with a predicted incidence rate of high sensory blocks closes to but less than one-half of the average incidence rate. Second, the upper boundary of the suggested dose was located at the dose with a predicted incidence rate of high sensory blocks close to but less than the average incidence rate.

The models’ performances were evaluated in terms of discrimination and calibration. All analyses were performed separately for each patient category. For discrimination, pairwise C-indexes using the conditional-risk method were estimated and reported [20]. For calibration, pairwise calibration plots contrasting the expected and observed probabilities were illustrated. To examine the potential utility of the suggested dose ranges in practice, we performed an apparent validation by comparing the incidence rates of achieving favorable sensory block levels and high SA between all patients and a group of patients who were administered with isobaric bupivacaine within the suggested dose ranges.

## 3. Results

**Figure 1:**
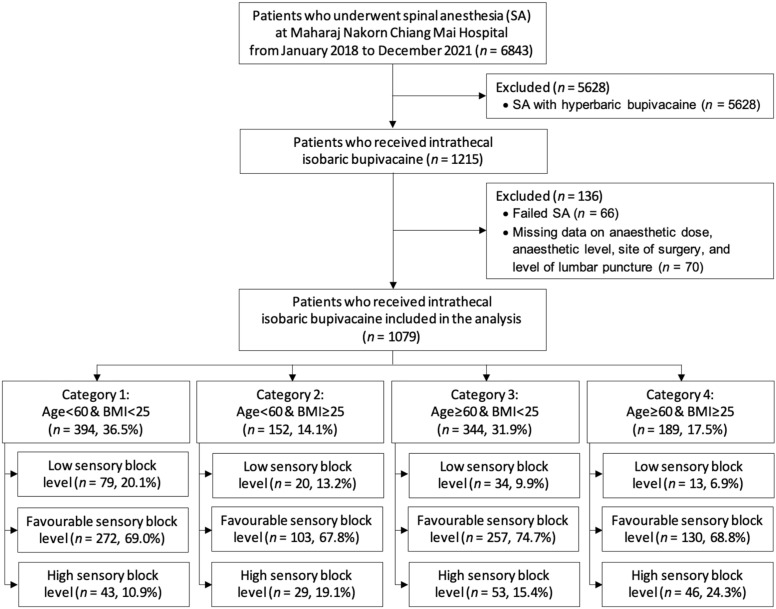
Study flow diagram.

During the study period, 6843 patients underwent SA. Of these, 5628 patients received intrathecal hyperbaric bupivacaine; 66 patients had failed SA (no sensory block tested); and 70 patients had missing data on the anesthetic dose, site of surgery, level of lumbar puncture, or sensory block level. Finally, 1079 patients were included in the analysis (Figure 1). Overall, 762 (70.6%) patients achieved a favorable block height, whereas the remaining 146 (13.5%) and 171 (15.9%) patients received low and high levels of sensory blocks, respectively. The mean age of the patients was 54.4 ± 20.6 years and the male/female ratio was almost equally distributed (47%:53%). There were significant differences in terms of the sex, age, weight, height, BMI, location of operation, and isobaric bupivacaine dose among the three groups of sensory block levels (Table 1). Table 1 shows the overall clinical characteristics of the study participants according to their sensory block height. The distribution of the peak sensory nerve block levels is also reported in Table 1.

All patients were divided into four categories according to age and BMI. The category with the highest proportion of patients was category 1 (age < 60 years and BMI < 25 kg/m^2^ (36.5%)), followed by category 3 (age ≥ 60 years and BMI < 25 kg/m^2^ (31.9%)), category 4 (age ≥ 60 years and BMI ≥ 25 kg/m^2^ (17.5%)), and category 2 (age < 60 years and BMI ≥ 25 kg/m^2^ (14.1%)). The incidence rate of high sensory blocks was the highest in category 4 (24.3%). Table 2 shows and compares the clinical characteristics of the study patients in each category across the three different groups of sensory block heights. The association between the isobaric bupivacaine dose and peak sensory block level was significant only in categories 1 and 2 (Table 2). However, a trend of increasing doses and higher sensory block levels was also observed in categories 3 and 4.

**Figure 2:**
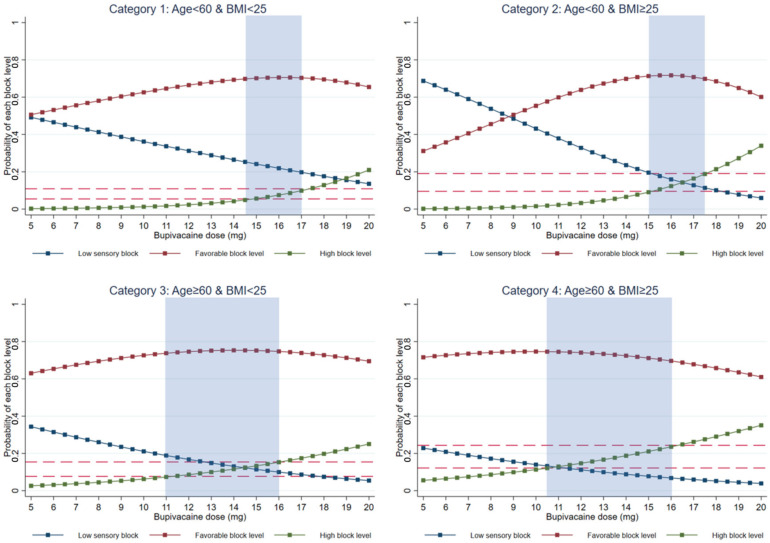
Marginal prediction curves visualizing the association between the isobaric bupivacaine dose and sensory block levels across 4 different categories of patients. The red solid line with square markers represents the predicted probabilities of achieving a favorable spinal block level. The blue solid line with square markers represents the predicted probabilities of achieving a low spinal block level, and the green solid line with square markers represents the predicted probabilities of achieving a high sensory block level after spinal anesthesia. The red upper dash line marks the average incidence rate of high sensory blocks, whereas the red lower dash line marks half the average incidence rate of high sensory blocks. The highlighted light-blue region represents the suggested range of isobaric bupivacaine doses for each subgroup.

Four multivariable polytomous logistic regression models were performed separately for each patient category (Supplementary Tables S1–S4), and marginal prediction curves were subsequently illustrated (Figure 2). The associations between the isobaric bupivacaine dose and the probability of achieving each group of sensory block height were similar for patients within the same age category, regardless of their BMI. Patients aged ≥60 years (categories 3 and 4) seemed to have a higher probability of achieving a favorable sensory block level with a lower bupivacaine dose, whereas the probability in patients aged <60 years (categories 1 and 2) was highest when a higher range of isobaric bupivacaine doses, at approximately 15 to 17 mg, was injected (Figure 2). Based on our pre-specified criteria, we provided recommendations for the suggested dose interval to achieve a favorable sensory block level while minimizing the incidence rate of high sensory blocks (Table 3). All four derived polytomous logistic models showed fair to excellent performance in discriminating between the three different groups of peak sensory block levels (Table 3), with acceptable calibration (Supplementary Figures S1–S4). When the overall patient data were compared with the selected data of patients who received isobaric bupivacaine within the suggested effective dose ranges, the overall incidence rate of achieving favorable sensory block levels increased from 70.6% to 73.9%, whereas the incidence rate of achieving high SA decreased from 15.9% to 12.1%. The improvement was more prominent in patients aged <60 years (categories 1 and 2) than in the older groups (categories 3 and 4). Table 4 presents the apparent validation results for each patient category.

## 4. Discussion

This study demonstrated that the sensory block height, whether low, favorable, or high, was significantly associated with an increasing dose of isobaric bupivacaine in patients aged <60 years, and this trend was observed in patients aged ≥60 years. Regardless of their BMI, the suggested dose ranges of isobaric bupivacaine to achieve a T5–T10 sensory block level with the potential to reduce the probability of high SA were approximately 15–17 and 10.5–16 mg for patients aged <60 and ≥60 years, respectively. At these dose ranges, the probability of achieving the T5–T10 sensory block level was approximately 74%. Therefore, these suggested doses are considered the ‘effective’ dose ranges for intrathecal isobaric bupivacaine.

Several factors influence the spread of an intrathecal local anesthetic. The patient characteristics, including the age, weight, height, BMI, sex, pregnancy, lumbosacral CSF volume, and abnormal spinal anatomy, are uncontrollable determinants, whereas the factors involving the spinal block technique, such as the site and speed of injection, orientation of the spinal needle tip, baricity and dose of local anesthetics, intrathecal adjuvant administration, and patient position, are adjustable and dependent on the anesthesiologist’s decision [3]. Without or with a minor impact from the gravitational force on an isobaric solution, the dosage of the local anesthetic is an important and controllable determinant of anesthetic spread [3,4]. The effective dose range of local anesthetics for successful and safe SA is wide in normal-sized adults and markedly narrower in pregnant women, the elderly, and obese patients, because these patient populations have a reduced lumbosacral CSF volume, which is a key patient-related determinant [8,9]. While effective doses (EDs) of intrathecal hyperbaric and isobaric bupivacaine for cesarean delivery have been comprehensively investigated by several research methods, dose–response studies specific to aging and obese populations have been reported scantly.

From the limited evidence, the EDs of intrathecal isobaric bupivacaine for elderly and increased BMI patients vary depending on the definition of success in each study. Van Egmond et al. demonstrated the median ED (ED50) and calculated ED95 of 0.5% isobaric bupivacaine in patients with an average age of 70 years to be 3.5 and 5 mg, respectively [21]. These EDs were adequate for unilateral total knee arthroplasty without a tourniquet and provided a duration of sensory and motor blockade of approximately 2 h. Chen et al. reported that the ED50s for 0.75% isobaric bupivacaine were 6.6 and 5.8 mg for 61- to 70- and 71- to 80-year-old patients, respectively [5]. These doses provided an analgesic level to T10-L1 at 10 min after spinal block and a duration of motor blockade of around 3–4 h. These previous studies reported noticeably smaller doses than ours, which suggested doses of 11–16 mg for patients aged ≥60 years. We opine that these discrepancies stemmed mainly from different research methodologies. While others studies were experimental studies aiming to find the median ED for restricted levels and durations of anesthesia, we took advantage of our observational data to identify the ED for more generalized circumstances that require a sensory block up to the T5–T10 spinal nerve levels. To achieve this favorable block height, our suggested dose interval was relatively consistent with the ED50 reported by Michalek-Sauberer et al. [22]. In this previous study, the ED50 of 0.5% isobaric bupivacaine was 11.2 mg for patients undergoing interstitial brachytherapy of the lower abdomen. Regarding the association between the BMI and dose of intrathecal isobaric bupivacaine, previous studies showed that an increased BMI was a determinant of the higher extent of SA and the dosing of isobaric bupivacaine was suggested to be inversely related to the BMI [11,12]. No dose–response study focusing on determining the ED of isobaric bupivacaine in overweight or obese non-pregnant patients has been reported. However, our study found that a dose reduction was not necessary for patients with BMI ≥ 25 kg/m^2^, regardless of their age. This finding indicates that when compared with the BMI, age is a dominant predictor of the intrathecal spread of isobaric bupivacaine.

The dose is a parameter dependent on the concentration and volume. Previous studies have reported conflicting effects of the dose and volume of intrathecal local anesthetics [3]. Some studies demonstrated greater intrathecal spread with a higher dose or volume of local anesthetics, and some showed no difference. With a fixed concentration of 0.5% isobaric bupivacaine in our study, a change in dose was accompanied by a proportionate change in the volume of administered bupivacaine. Therefore, whether the dose or volume plays more role in determining the sensory block height could not be differentiated in our study.

The methodology of our study deserves discussion. We introduced a logical approach to investigate the ED of intrathecal isobaric bupivacaine. The estimation of the suggested dose intervals in our study was based on the rationale that generously accepted sensory blocks as high as the T5 level but strictly avoided sensory block levels ≥ T4 (i.e., high SA). In clinical practice, even though the trend towards ambulatory surgery and early ambulation is currently widespread, several ordinary operations increase surgical complexities, such as a revision hip replacement, which require extended operation times [23]. This situation is challenging for anesthesiologists to decide how much isobaric bupivacaine should be administered to provide successful and safe SA without retaining the spinal or epidural catheter. We, therefore, set the pre-specified criteria particularly related to the predicted incidence rate of high SA in each patient category. In addition, an observational design provides benefits in terms of generalizability in comparison to conventional dose-finding experimental studies. For instance, the level of the lumbar puncture, which has been reported as a determinant affecting the block height, was selected at the anesthesiologists’ discretion and could not be limited to one level in our study [24,25]. This made our results valid irrespective of the level of local anesthetic injection that might be incorrectly identified by palpation [26].

### Limitations

This study has some limitations. First, in addition to the extent of the sensory block, an assessment of the motor block levels and durations of anesthesia should also be incorporated to demonstrate the clinically effective doses of intrathecal local anesthetics because the dose directly affects the anesthetic duration, particularly in aging and obese patients [27,28]. Second, clinically relevant outcomes following high SA or inadequate SA, such as hypotension, bradycardia, or conversion to general anesthesia, were not recorded in this study. The association between these unfavorable events and the anesthetic height, as well as the dose of bupivacaine, should be further specified. Third, it is the nature of a retrospective observational study that could not control for potential confounding factors (e.g., level of lumbar puncture, and size of spinal needle) that might mask the actual association between the dose of isobaric bupivacaine and the anesthetic level. However, this study design reflects pragmatic practice and has good generalizability. Finally, when the suggested dose ranges were applied to the dataset, the proportion of achieving the favorable sensory block levels increased minimally and that of the high block levels reduced modestly. This point may raise concerns regarding the clinical utility of our findings. However, this could be explained by the distribution of the bupivacaine dose, which leaned toward the upper boundary of the suggested dose ranges in all patient subcategories. If the average bupivacaine doses were lower and close to the lower boundary, we would observe a significant reduction in high SA while preserving the incidence rate of favorable block levels > 70%. Further studies are warranted to validate the clinical utility of our suggested effective bupivacaine dose range.

## 5. Conclusions

The effective dose ranges of 0.5% isobaric bupivacaine to achieve T5–T10 sensory block levels were 15–17 and 10.5–16 mg for patients aged <60 and ≥60 years, respectively. In other words, the effective volumes of 0.5% isobaric bupivacaine were 3.0–3.4 and 2.1–3.2 mL for patients aged <60 and ≥60 years, respectively. Within these dose (or volume) intervals, a higher dose (or volume) increased the probability of high SA, but not at levels greater than 10 to 15%. Conversely, a smaller dose (or volume) increased the probability of a lower sensory block level. Therefore, the suggested doses could guide anesthesiologists to select a safe and effective dose of intrathecal isobaric bupivacaine for aging and increased BMI patients. Further prospective dose–response studies are encouraged to determine the ED50 and ED95 values of isobaric bupivacaine in these specific populations.

## Figures and Tables

**Table 1:** Overall clinical characteristics of the study patients by sensory block levels.

Characters	Low Sensory Block Level (≤T11)		Favorable Sensory Block Level (T5–T10)		High Sensory Block Level (≥T4)		*p* Value
	(*n* = 146)		(*n* = 762)		(*n* = 171)		
Male	89	(61.0)	366	(48.0)	62	(36.3)	<0.001
Age (year)	45.8	±19.9	55.1	±20.7	58.5	±18.5	<0.001
Weight (kg)	61.2	±10.4	60.1	±12.4	63.0	±12.5	0.016
Height (cm)	163.6	±8.2	160.1	±9.3	158.7	±9.1	<0.001
BMI (kg/m^2^)	22.9	±3.4	23.4	±4.1	25.0	±4.5	<0.001
Location of operation							
Upper abdomen	0	(0)	1	(0.1)	0	(0)	0.025
Lower abdomen	0	(0)	4	(0.5)	2	(1.2)	
Groin/inguinal	1	(0.7)	24	(3.2)	1	(0.6)	
Hip	11	(7.5)	85	(11.2)	10	(5.9)	
Lower extremity	127	(87.0)	586	(76.9)	152	(88.9)	
Perineum/anus	1	(0.7)	24	(3.2)	2	(1.2)	
Others	6	(4.1)	38	(5.0)	4	(2.3)	
Injection level							
L2–L3	8	(5.5)	31	(4.1)	7	(4.1)	0.253
L3–L4	98	(67.1)	531	(69.7)	118	(69.0)	
L4–L5	36	(24.7)	194	(25.5)	46	(26.9)	
L5–S1	4	(2.7)	6	(0.8)	0	(0)	
Bupivacaine dose (mg)	16.1	±2.5	16.4	±2.3	17.2	±2.3	<0.001
Peak level of sensory nerve block							
T1–T2	-	-	-	-	9	(5.3)	<0.001
T3–T4	-	-	-	-	162	(94.7)	
T5–T6	-	-	215	(28.2)	-	-	
T7–T8	-	-	183	(24.0)	-	-	
T9–T10	-	-	364	(47.8)	-	-	
T11–T12	113	(77.4)	-	-	-	-	
L1–L2	33	(22.6)	-	-	-	-	

Values are presented as numbers (%) or means ± SD; *p* values were tested using a one-way analysis of variance (ANOVA) for continuous variables and Fisher’s exact probability test or Chi-square test for categorical variables, as appropriate. BMI: body mass index.

**Table 2:** Clinical characteristics of the study patients categorized according to age and body mass index compared across sensory block levels.

Characters	Low Sensory Block Level (≤T11)		Favorable Sensory Block Level (T5–T10)		High Sensory Block Level (≥T4)		*p* Value
Category 1 (*n* = 394)	79	(20.1)	272	(69.0)	43	(10.9)	
Male	54	(68.4)	168	(61.8)	24	(55.8)	0.357
Age (year)	34.4	±14.1	37.0	±15.2	39.6	±14.2	0.163
Weight (kg)	59.1	±9.5	57.0	±8.4	57.4	±8.6	0.163
Height (cm)	165.2	±8.6	163.3	±8.8	162.9	±8.1	0.194
BMI (kg/m^2^)	21.6	±2.4	21.3	±2.2	21.6	±2.2	0.559
Injection level							
L2–L3	5	(6.3)	9	(3.3)	1	(2.3)	0.406
L3–L4	53	(67.1)	192	(70.6)	29	(67.4)	
L4–L5	20	(25.3)	71	(26.1)	13	(30.2)	
L5–S1	1	(1.3)	0	(0)	0	(0)	
Bupivacaine dose (mg)	16.4	±2.6	16.8	±2.1	18.0	±2.1	<0.001
Category 2 (*n* = 152)	20	(13.2)	103	(67.8)	29	(19.1)	
Male	13	(65.0)	67	(65.1)	16	(55.2)	0.622
Age (year)	37.8	±13.4	40.9	±12.6	43.7	±14.5	0.295
Weight (kg)	72.6	±5.7	76.5	±10.6	77.4	±12.1	0.244
Height (cm)	161.8	±7.9	164.6	±9.6	162.2	±11.5	0.317
BMI (kg/m^2^)	27.8	±1.7	28.2	±2.7	29.5	±4.7	0.082
Injection level							
L2–L3	1	(5.0)	3	(2.9)	0	(0)	0.236
L3–L4	13	(65.0)	75	(72.8)	22	(75.9)	
L4–L5	5	(25.0)	25	(24.3)	7	(24.1)	
L5–S1	1	(5.0)	0	(0)	0	(0)	
Bupivacaine dose (mg)	16.0	±2.1	17.2	±2.2	18.2	±1.8	0.003
Category 3 (*n* = 344)	34	(9.9)	257	(74.7)	53	(15.4)	
Male	18	(52.9)	99	(38.5)	12	(22.6)	0.013
Age (year)	68.5	±8.6	73.0	±9.6	72.1	±8.2	0.028
Weight (kg)	56.8	±9.7	52.5	±9.1	53.9	±8.5	0.031
Height (cm)	162.7	±6.8	157.2	±8.6	155.3	±8.8	<0.001
BMI (kg/m^2^)	21.4	±3.0	21.2	±2.5	22.3	±2.2	0.014
Injection level							
L2–L3	2	(5.9)	11	(4.3)	2	(3.8)	0.947
L3–L4	22	(64.7)	170	(66.2)	35	(66.0)	
L4–L5	10	(29.4)	72	(28.0)	16	(30.2)	
L5–S1	0	(0)	4	(1.6)	0	(0)	
Bupivacaine dose (mg)	15.7	±2.7	15.7	±2.4	16.5	±2.7	0.094
Category 4 (*n* = 189)	13	(6.9)	130	(68.8)	46	(24.3)	
Male	4	(30.8)	32	(24.6)	10	(21.7)	0.750
Age (year)	68.1	±6.0	69.0	±7.3	69.6	±7.8	0.786
Weight (kg)	68.2	±6.3	68.4	±8.7	69.5	±6.6	0.712
Height (cm)	158.6	±6.6	155.8	±7.5	156.5	±5.8	0.347
BMI (kg/m^2^)	27.1	±1.4	28.2	±3.0	28.4	±2.6	0.309
Injection level							
L2–L3	0	(0)	8	(6.2)	4	(8.7)	0.026
L3–L4	10	(76.9)	94	(72.3)	32	(69.6)	
L4–L5	1	(7.7)	26	(20.0)	10	(21.7)	
L5–S1	2	(15.4)	2	(1.5)	0	(0)	
Bupivacaine dose (mg)	15.7	±2.1	16.1	±2.1	16.6	±2.0	0.196

Values are presented as numbers (%) or means ± SD. Category 1: age < 60 and BMI < 25; category 2: age < 60 and BMI ≥ 25; category 3: age ≥ 60 and BMI < 25; category 4: age ≥ 60 and BMI ≥ 25. *p* values were tested using a one-way analysis of variance (ANOVA) for continuous variables and Fisher’s exact probability test or Chi-square test for categorical variables, as appropriate. BMI: body mass index.

**Table 3:** Suggested range of isobaric bupivacaine dosages for each patient category based on the predicted curves of polytomous logistic regression models and their discriminative ability.

	Category 1: Age < 60 BMI < 25	Category 2: Age < 60 BMI ≥ 25	Category 3: Age ≥ 60 BMI < 25	Category 4: Age ≥ 60 BMI ≥ 25
Suggested dose range for favorable sensory block level (mg)	14.5–17.0	15.0–17.5	11.0–16.0	10.5–16.0
Pairwise c-indexes				
Low vs. Favorable	0.62 (0.55, 0.69)	0.73 (0.62, 0.84)	0.75 (0.66, 0.83)	0.81 (0.73, 0.90)
Low vs. High	0.74 (0.65, 0.83)	0.84 (0.74, 0.95)	0.82 (0.73, 0.90)	0.86 (0.75, 0.97)
Favorable vs. High	0.67 (0.59, 0.76)	0.66 (0.53, 0.79)	0.68 (0.60, 0.77)	0.62 (0.53, 0.72)

Values are presented as min–max or pairwise c-indexes (95% confidence interval). BMI: body mass index.

**Table 4:** Apparent validation of the suggested effective dose range in achieving favorable and high sensory blocks.

	Frequency and Percentage of High Sensory Block			Frequency and Percentage of Favorable Sensory Block		
	Total	Within Suggested Dose Range	Out of Suggested Dose Range	Total	Within Suggested Dose Range	Out of Suggested Dose Range
Overall	171/1079 (15.9)	69/571 (12.1)	102/508 (20.1)	762/1079 (70.6)	422/571 (73.9)	340/508 (66.9)
Dose (mg)	16.5 ± 2.4	15.1 ± 1.3	18.0 ± 2.3	16.5 ± 2.4	15.1 ± 1.3	18.0 ± 2.3
Category 1	43/394 (10.9)	14/191 (7.3)	29/203 (14.3)	272/394 (69.0)	140/191 (73.3)	132/203 (65.0)
Dose (mg)	16.9 ± 2.3	15.7 ± 0.8	18 ± 2.6	16.9 ± 2.3	15.7 ± 0.8	18 ± 2.6
Category 2	29/152 (19.1)	8/75 (10.7)	21/77 (27.3)	103/152 (67.8)	55/75 (73.3)	48/77 (62.3)
Dose (mg)	17.2 ± 2.2	16.0 ± 1.0	18.4 ± 2.5	17.2 ± 2.2	16.0 ± 1.0	18.4 ± 2.5
Categories 1 and 2	72/546 (13.2)	22/266 (8.3)	50/280 (17.9)	375/546 (68.7)	195/266 (73.3)	180/280 (64.3)
Dose (mg)	17.0 ± 2.2	15.8 ± 0.9	18.1 ± 2.6	17.0 ± 2.2	15.8 ± 0.9	18.1 ± 2.6
Category 3	53/344 (15.4)	24/199 (12.1)	29/145 (20.0)	257/344 (74.7)	153/199 (76.9)	104/145 (71.7)
Dose (mg)	15.8 ± 2.5	14.3 ± 1.3	17.9 ± 2.2	15.8 ± 2.5	14.3 ± 1.3	17.9 ± 2.2
Category 4	46/189 (24.3)	23/106 (21.7)	23/83 (27.7)	130/189 (68.8)	74/106 (69.8)	56/83 (67.5)
Dose (mg)	16.2 ± 2.1	14.8 ± 1.0	17.9 ± 1.7	16.2 ± 2.1	14.8 ± 1.0	17.9 ± 1.7
Categories 3 and 4	99/533 (18.6)	47/305 (15.4)	52/228 (22.8)	387/533 (72.6)	227/305 (74.4)	160/228 (70.2)
Dose (mg)	16.0 ± 2.3	14.5 ± 1.2	17.9 ± 2.1	16.0 ± 2.3	14.5 ± 1.2	17.9 ± 2.1

Values are presented as numbers (%) or means ± SD. Category 1: age < 60 and BMI < 25; category 2: age < 60 and BMI ≥ 25; category 3: age ≥ 60 and BMI < 25; category 4: age ≥ 60 and BMI ≥ 25. BMI: body mass index.

## Data Availability

The data presented in this study are available in the article and Supplementary Materials.

## Supplementary Materials

The following supporting information can be downloaded at: https://www.mdpi.com/article/10.3390/medicina59030484/s1. Supplementary Figure S1: Discriminative ability based on the area under the receiver operating characteristics curves (a–c) and calibration plots (d–f) for the polytomous logistic model in patients aged less than 60 years old with a body mass index of less than 25 kg/m^2^ (category 1). Supplementary Figure S2: Discriminative ability based on the area under the receiver operating characteristics curves (a–c) and calibration plots (d–f) for the polytomous logistic model in patients aged less than 60 years old with a body mass index of more than or equal to 25 kg/m^2^ (category 2). Supplementary Figure S3: Discriminative ability based on the area under the receiver operating characteristics curves (a–c) and calibration plots (d–f) for the polytomous logistic model in patients aged more than or equal to 60 years old with a body mass index of less than 25 kg/m^2^ (category 3). Supplementary Figure S4: Discriminative ability based on the area under the receiver operating characteristics curves (a–c) and calibration plots (d–f) for the polytomous logistic model in patients aged more than or equal to 60 years old with a body mass index of more than or equal to 25 kg/m^2^ (category 4). Supplementary Table S1: Multivariable polytomous logistic regression model in patients aged less than 60 years old with a body mass index of less than 25 kg/m^2^ (category 1). Supplementary Table S2: Multivariable polytomous logistic regression model in patients aged less than 60 years old with a body mass index of more than or equal to 25 kg/m^2^ (category 2). Supplementary Table S3: Multivariable polytomous logistic regression model in patients aged more than or equal to 60 years old with a body mass index of less than 25 kg/m^2^ (category 3). Supplementary Table S4: Multivariable polytomous logistic regression model patients aged more than or equal to 60 years old with a body mass index of more than or equal to 25 kg/m^2^ (category 4).
