# Supplementary material for: Effective Dose Range of Intrathecal Isobaric Bupivacaine to Achieve T5–T10 Sensory Block Heights for Elderly and Overweight Patients: An Observational Study
Source: Medicina (Kaunas). 2023 Mar 1;59(3):484. doi: 10.3390/medicina59030484 (PMC10057130; doi:10.3390/medicina59030484)
Supplement: Supplementary file 1 [file medicina-59-00484-s001.zip › medicina-2230908-supplementary.pdf]

SUPPLEMENTARY FIGURES AND TABLES

Effective Dose of Intrathecal Isobaric Bupivacaine to Achieve T5-T10 Block Height for Aged and Overweight Patients

TITLES OF FIGURES

Supplementary Figure S1 — Discriminative ability based on the area under the receiver operating characteristics curves (a-c) and calibration plots (d-f) for the polytomous logistic model in patients aged less than 60 years old with body mass index less than 25 kg/m^2 (Category 1).

Supplementary Figure S2 — Discriminative ability based on the area under the receiver operating characteristics curves (a-c) and calibration plots (d-f) for the polytomous logistic model in patients aged less than 60 years old with body mass index more than or equal to 25 kg/m^2 (Category 2).

Supplementary Figure S3 — Discriminative ability based on the area under the receiver operating characteristics curves (a-c) and calibration plots (d-f) for the polytomous logistic model in patients aged more than or equal to 60 years old with body mass index less than 25 kg/m^2 (Category 3).

Supplementary Figure S4 — Discriminative ability based on the area under the receiver operating characteristics curves (a-c) and calibration plots (d-f) for the polytomous logistic model in patients aged more than or equal to 60 years old with body mass index more than or equal to 25 kg/m^2 (Category 4).

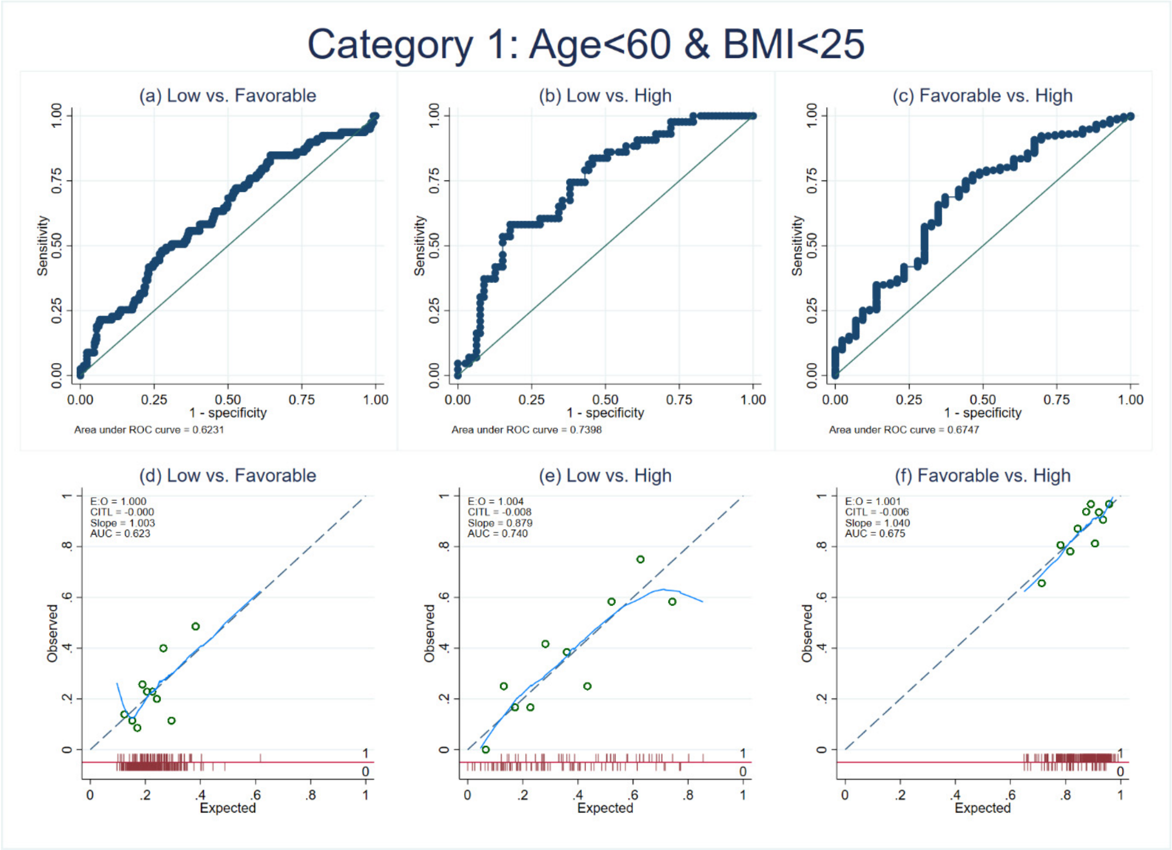

Supplementary Figure S1 — Discriminative ability based on the area under the receiver operating characteristics curves (a-c) and calibration plots (d-f) for the polytomous logistic model in patients aged less than 60 years old with body mass index less than 25 kg/m^2 (Category 1).

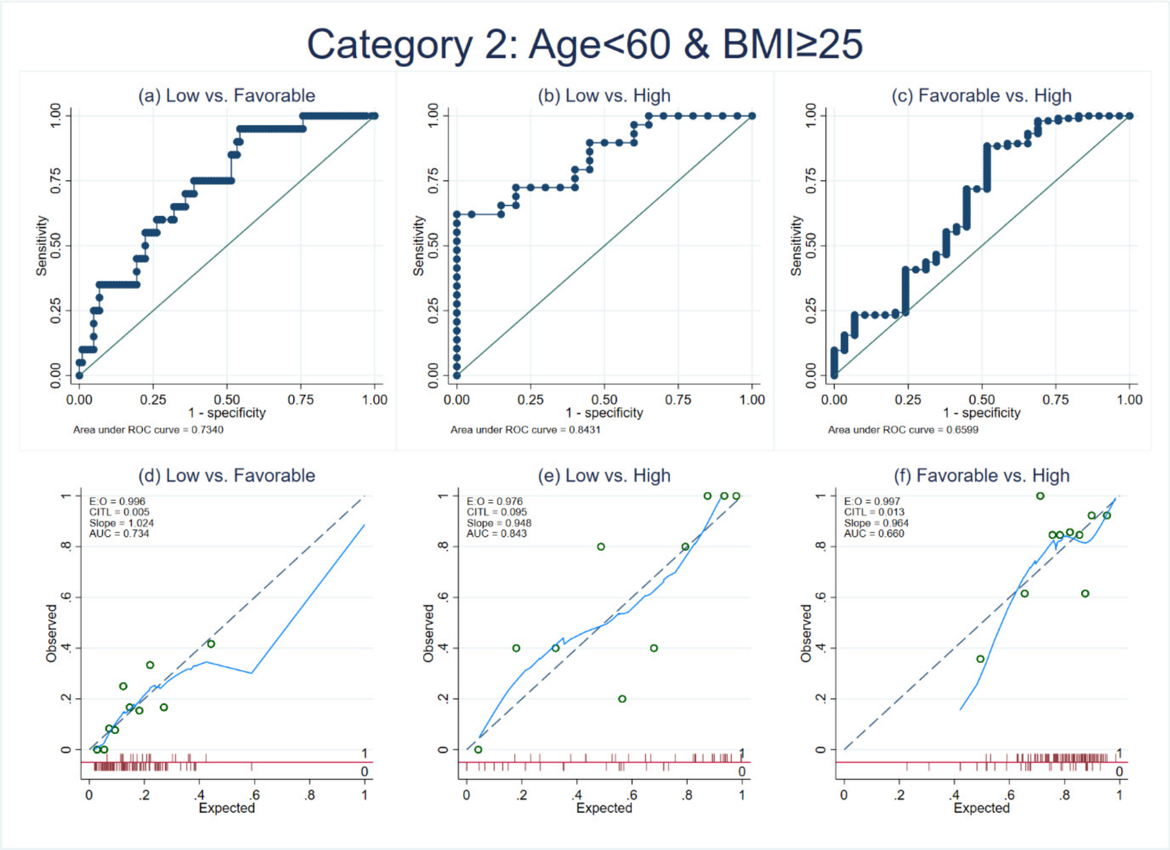

Supplementary Figure S2 — Discriminative ability based on the area under the receiver operating characteristics curves (a-c) and calibration plots (d-f) for the polytomous logistic model in patients aged less than 60 years old with body mass index more than or equal to 25 kg/m^2 (Category 2).

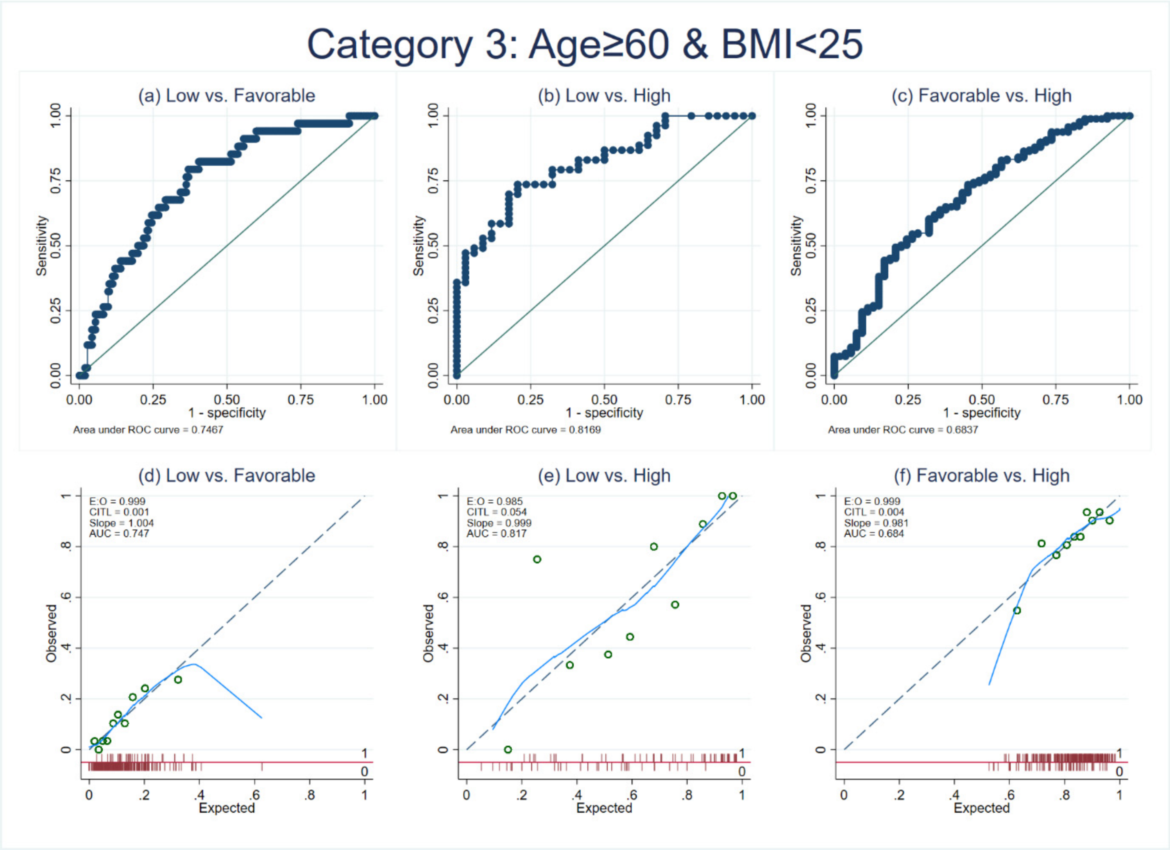

Supplementary Figure S3 — Discriminative ability based on the area under the receiver operating characteristics curves (a-c) and calibration plots (d-f) for the polytomous logistic model in patients aged more than or equal to 60 years old with body mass index less than 25 kg/m^2 (Category 3).

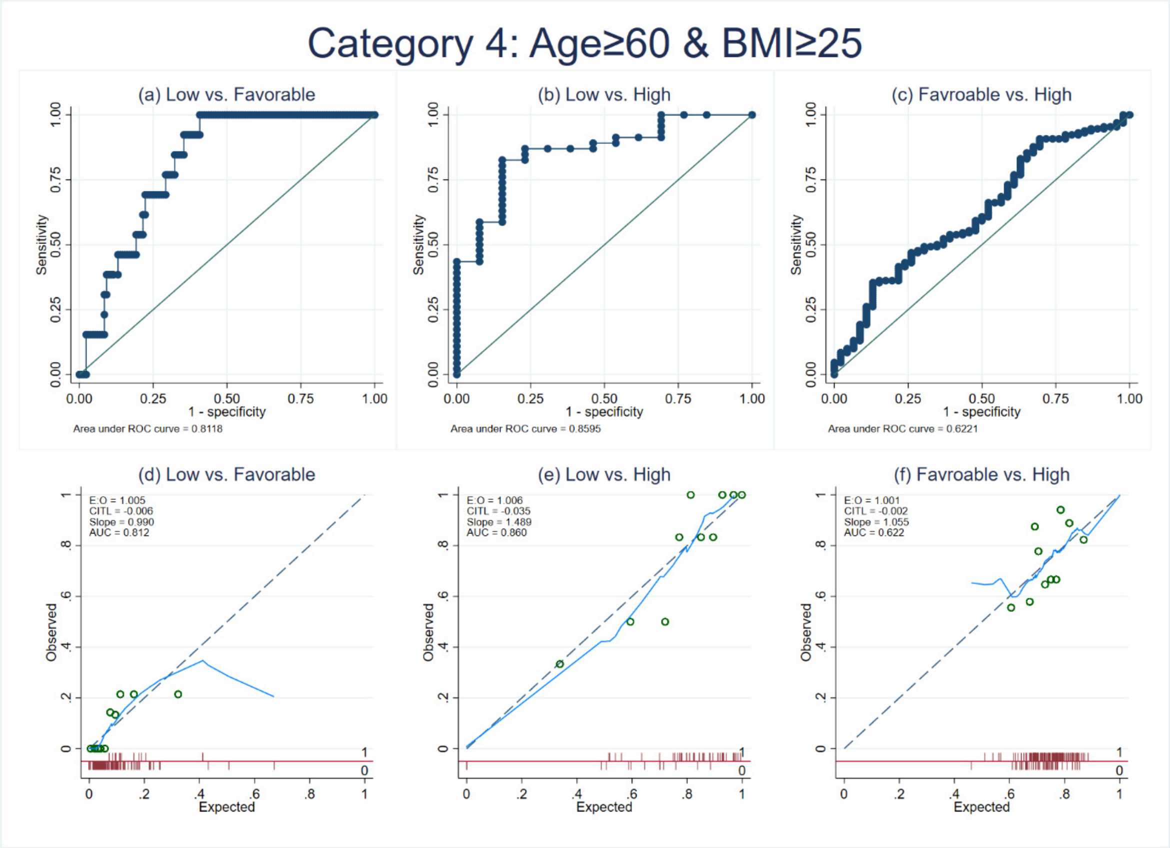

Supplementary Figure S4 — Discriminative ability based on the area under the receiver operating characteristics curves (a-c) and calibration plots (d-f) for the polytomous logistic model in patients aged more than or equal to 60 years old with body mass index more than or equal to 25 kg/m^2 (Category 4).

TABLES

Supplementary Table S1 — Multivariable polytomous logistic regression model in patients aged less than 60 years old with body mass index less than 25 kg/m^2 (Category 1).

Supplementary Table S2 — Multivariable polytomous logistic regression model in patients aged less than 60 years old with body mass index more than or equal to 25 kg/m^2 (Category 2).

Supplementary Table S3 — Multivariable polytomous logistic regression model in patients aged more than or equal to 60 years old with body mass index less than 25 kg/m^2 (Category 3).

Supplementary Table S4 — Multivariable polytomous logistic regression model patients aged more than or equal to 60 years old with body mass index more than or equal to 25 kg/m^2 (Category 4).

Supplementary Table S1 — Multivariable polytomous logistic regression model in patients aged less than 60 years old with body mass index less than 25 kg/m^2 (Category 1).

Predictors	Beta coefficient	95% confidence interval	P-value
**Lower sensory block level**			
Isobaric bupivacaine dose (Each 1-mg increase)	-0.1076047	-0.2240038, 0.0087944	0.070
Male sex	0.0536703	-0.5959009, 0.7032415	0.871
Age of the patient (Each 1-year increase)	-0.0127394	-0.0316219, 0.006143	0.186
Height (Each 1-cm increase)	0.0020543	-0.0447476, 0.0488562	0.931
Weight (Each 1-kg increase)	0.0263514	-0.015996, 0.0686989	0.223
Injection level			
L2-L3	Reference		
L3-L4	-0.6308244	-1.802002, 0.5403528	0.291
L4-L5	-0.5783501	-1.808458, 0.6517575	0.357
L5-S1	14.16603	-1699.995, 1728.327	0.987
Constant (intercept)	-0.2122009	-7.491522, 7.06712	0.954
**Favorable sensory block level**			
	(Base outcome)		
**Higher sensory block level**			
Isobaric bupivacaine dose (Each 1-mg increase)	0.2804427	0.1149919, 0.4458935	0.001
Male sex	-0.4363014	-1.266455, 0.3938524	0.303
Age of the patient (Each 1-year increase)	0.008804	-0.0144479, 0.0320559	0.458
Height (Each 1-cm increase)	-0.0154071	-0.0801769, 0.0493627	0.641
Weight (Each 1-kg increase)	0.0175183	-0.0408801, 0.0759167	0.557
Injection level			
L2-L3	Reference		
L3-L4	0.180821	-1.958781, 2.320423	0.868
L4-L5	0.4450736	-1.742352, 2.6325	0.690
L5-S1	-0.0286841	-4639.541, 4639.484	1.000
Constant (intercept)	-6.437252	-16.40996, 3.535454	0.206

Supplementary Table S2 — Multivariable polytomous logistic regression model in patients aged less than 60 years old with body mass index more than or equal to 25 kg/m^2 (Category 2).

Predictors	Beta coefficient	95% confidence interval	P-value
**Lower sensory block level**			
Isobaric bupivacaine dose (Each 1-mg increase)	-0.237644	-0.4809599, 0.0056719	0.056
Male sex	0.9469826	-0.4154881, 2.309453	0.173
Age of the patient (Each 1-year increase)	-0.0389472	-0.0882352, 0.0103408	0.121
Height (Each 1-cm increase)	-0.0392374	-0.140525, 0.0620501	0.448
Weight (Each 1-kg increase)	-0.0439194	-0.1284941, 0.0406554	0.309
Injection level			
L2-L3	Reference		
L3-L4	-0.5716221	-3.374747, 2.231503	0.689
L4-L5	-0.3214876	-3.261515, 2.61854	0.830
L5-S1	20.35857	-36462.35, 36503.06	0.999
Constant (intercept)	15.22241	-1.16929, 31.61412	0.080
**Favorable sensory block level**			
	(Base outcome)		
**Higher sensory block level**			
Isobaric bupivacaine dose (Each 1-mg increase)	0.3250955	0.0826547, 0.5675362	0.009
Male sex	-0.2471345	-1.408985, 0.9147157	0.677
Age of the patient (Each 1-year increase)	0.0095255	-0.0270956, 0.0461465	0.610
Height (Each 1-cm increase)	-0.0620276	-0.1373253, 0.0132702	0.106
Weight (Each 1-kg increase)	0.0483253	-0.0065498, 0.1032004	0.084
Injection level			
L2-L3	Reference		
L3-L4	14.65271	-1894.947, 1924.252	0.988
L4-L5	14.63849	-1894.961, 1924.238	0.988
L5-S1	15.0252	-77843.41, 77873.46	1.000
Constant (intercept)	-15.95827	-1925.597, 1893.681	0.987

Supplementary Table S3 — Multivariable polytomous logistic regression model in patients aged more than or equal to 60 years old with body mass index less than 25 kg/m^2 (Category 3).

Predictors	Beta coefficient	95% confidence interval	P-value
**Lower sensory block level**			
Isobaric bupivacaine dose (Each 1-mg increase)	-0.1493778	-0.3254235, 0.0266679	0.096
Male sex	-0.0294145	-0.9073335, 0.8485045	0.948
Age of the patient (Each 1-year increase)	-0.061157	-0.1105168, -0.0117972	0.015
Height (Each 1-cm increase)	0.0931226	0.0230627, 0.1631825	0.009
Weight (Each 1-kg increase)	-0.0178304	-0.0737118, 0.0380511	0.532
Injection level			
L2-L3	Reference		
L3-L4	-0.0886829	-1.828916, 1.651551	0.920
L4-L5	-0.1425914	-1.945926, 1.660743	0.877
L5-S1	-14.77973	-3197.059, 3167.5	0.993
Constant (intercept)	-9.150924	-19.257, 0.9551479	0.076
**Favorable sensory block level**			
	(Base outcome)		
**Higher sensory block level**			
Isobaric bupivacaine dose (Each 1-mg increase)	0.1535893	0.0164925, 0.2906861	0.028
Male sex	-0.661633	-1.477977, 0.1547112	0.112
Age of the patient (Each 1-year increase)	0.0108957	-0.025998, 0.0477895	0.563
Height (Each 1-cm increase)	-0.0803621	-0.1425656, -0.0181586	0.011
Weight (Each 1-kg increase)	0.078512	0.0194528, 0.1375713	0.009
Injection level			
L2-L3	Reference		
L3-L4	0.0403298	-1.552061, 1.632721	0.960
L4-L5	0.178146	-1.469807, 1.826099	0.832
L5-S1	-14.32359	-2837.696, 2809.049	0.992
Constant (intercept)	3.655893	-4.806349, 12.11813	0.397

Supplementary Table S4 — Multivariable polytomous logistic regression model patients aged more than or equal to 60 years old with body mass index more than or equal to 25 kg/m^2 (Category 4).

Predictors	Beta coefficient	95% confidence interval		P-value
**Lower sensory block level**				
Isobaric bupivacaine dose	-.127783	-.4601431	.2045771	0.451
(Each 1-mg increase)				
Male sex	-.1399346	-1.720711	1.440842	0.862
Age of the patient	-.0301321	-.1243892	.064125	0.531
(Each 1-year increase)				
Height	.1460785	-.0008254	.2929824	0.051
(Each 1-cm increase)				
Weight	-.1001937	-.2402288	.0398414	0.161
(Each 1-kg increase)				
Injection level				
L2-L3	Reference			
L3-L4	12.87662	-1377.775	1403.528	0.986
L4-L5	11.79574	-1378.857	1402.449	0.987
L5-S1	15.50167	-1375.151	1406.155	0.983
Constant (intercept)	-27.15596	-1417.948	1363.636	0.969
**Favorable sensory block level**				
	(Base outcome)			
**Higher sensory block level**				
Isobaric bupivacaine dose	.1364955	-.0315812	.3045721	0.111
(Each 1-mg increase)				
Male sex	-.4004332	-1.357776	.5569093	0.412
Age of the patient	.0274078	-.0219869	.0768024	0.277
(Each 1-year increase)				
Height	.0212601	-.0455438	.088064	0.533
(Each 1-cm increase)				
Weight	.0151518	-.0373299	.0676335	0.571
(Each 1-kg increase)				
Injection level				
L2-L3	Reference			
L3-L4	-.2188682	-1.517257	1.079521	0.741
L4-L5	-.1969478	-1.624356	1.23046	0.787
L5-S1	-25.38965	-687154.6	687103.8	1.000
Constant (intercept)	-9.22818	-20.43689	1.980526	0.107
